# Elevating H3K27me3 level sensitizes colorectal cancer to oxaliplatin

**DOI:** 10.1093/jmcb/mjz032

**Published:** 2019-05-08

**Authors:** Qi Wang, Xi Chen, Yuhang Jiang, Sanhong Liu, Hanshao Liu, Xiaohua Sun, Haohao Zhang, Zhi Liu, Yu Tao, Cuifeng Li, Yiming Hu, Dandan Liu, Deji Ye, Yongzhong Liu, Mingliang Wang, Xiaoren Zhang

**Affiliations:** 1 The Key Laboratory of Stem Cell Biology, Shanghai Jiao Tong University School of Medicine & Shanghai Institutes for Biological Sciences, Chinese Academy of Sciences, Shanghai 200025, 227 Chongqing South Road, Shanghai, China; 2 Affiliated Cancer Hospital & Institute, Guangzhou Medical University, Guangzhou 510000, 195 Dongfeng West Road, Guangzhou, China; 3 Shanghai Institute for Advanced Immunochemical Studies, ShanghaiTech University, Shanghai 201210, 393 Huaxia Middle Road, Shanghai, China; 4 State Key Laboratory of Oncogenes and Related Genes, Shanghai Cancer Institute, Renji Hospital, Shanghai Jiao Tong University School of Medicine, Shanghai 200032, Ruijin 2nd Road, Shanghai, China; 5 Department of General Surgery, Ruijin Hospital, Shanghai Jiao Tong University School of Medicine, Shanghai 200025, Ruijin 2nd Road, Shanghai, China

**Keywords:** H3K27 trimethylation, colorectal cancer, chemoresistance, NOTCH signaling

## Abstract

Histone methylation is a context-dependent modification that regulates gene expression, and the trimethylation of histone H3 lysine 27 (H3K27me3) usually induces gene silencing. Overcoming colorectal cancer (CRC) chemoresistance is currently a huge challenge, but the relationship between H3K27me3 modification and chemoresistance remains largely unclear. Here, we found that H3K27me3 levels positively correlated with the metastasis-free survival of CRC patients and a low H3K27me3 level predicted a poor outcome upon chemotherapeutic drug treatment. Oxaliplatin stimulation significantly induced the expression of H3K27 lysine demethylase 6A/6B (KDM6A/6B), thus decreasing the level of H3K27me3 in CRC cells. Elevation of H3K27me3 level through KDM6A/6B depletion or GSK-J4 (a KDM6A/6B inhibitor) treatment significantly enhanced oxaliplatin-induced apoptosis. Conversely, when inhibiting the expression of H3K27me3 by EPZ-6438, an inhibitor of the histone methyltransferase EZH2, the proportion of apoptotic cells remarkably decreased. In addition, the combination of GSK-J4 and oxaliplatin significantly inhibited tumor growth in an oxaliplatin-resistant patient-derived xenograft model. Importantly, we revealed that oxaliplatin treatment dramatically induced NOTCH2 expression, which was caused by downregulation of H3K27me3 level on the NOTCH2 transcription initiation site. Thus, the activated NOTCH signaling promoted the expression of stemness-related genes, which resulted in oxaliplatin resistance. Furthermore, oxaliplatin-induced NOTCH signaling could be interrupted by GSK-J4 treatment. Collectively, our findings suggest that elevating H3K27me3 level can improve drug sensitivity in CRC patients.

## Introduction

Colorectal cancer (CRC) ranks third in morbidity and fourth in mortality of all human cancers worldwide (Fearon, 2011). The incidence of CRC in China has increased remarkably coinciding with the improvement of living standards over the past decade (Zhou et al., 2015). Oxaliplatin, a third-generation platinum compound, is generally considered to be a first-line chemotherapy for CRC patients (Meyerhardt and Mayer, 2005). However, CRC patients frequently develop chemoresistance and subsequently fail oxaliplatin treatment (Goldberg et al., 2004).

Hypothetical drug resistance mechanisms include inefficient cellular drug accumulation (Martinez-Balibrea et al., 2015), increased DNA repair (Ahmad, 2010; Abu-Zhayia et al., 2018), enhancement of anti-apoptosis pathways (Gourdier et al., 2002; Huang and Hung, 2009), and activation of the antioxidant glutathione system for detoxification (Sau et al., 2010). Recent research suggests that CRC contains cancer stem cells (CSCs), which act as the main player for tumor chemoresistance (Dean et al., 2005; O’Brien et al., 2007; Maugeri-Sacca et al., 2011). Multiple cell surface proteins are considered as markers of colorectal CSCs, including CD44, CD133, Lgr5, EpCAM, and ALDH (Dalerba et al., 2007; Huang, 2009; Kemper et al., 2012; Todaro et al., 2014). Numerous signaling pathways are involved in regulating CSCs, including the Hedgehog pathway, the NOTCH pathway, the NF-κB pathway, the PI3K/Akt pathway, and the Wnt/β-catenin pathway (Takebe et al., 2011; Matsui, 2016). NOTCH signal maintains the stemness of CSCs by promoting the expression of downstream target genes (Pannuti et al., 2010; Long et al., 2018). CD133, Sox2, and Oct4 are NOTCH target genes that are closely related to tumor initiation and colorectal CSC formation (Singh et al., 2003; Ricci-Vitiani et al., 2007). At the same time, continuous chemotherapeutic drug treatment also activates the NOTCH signaling pathway (Liau et al., 2017). Accordingly, we propose that targeting the NOTCH signaling pathway and inhibiting colorectal CSCs may be an effective method to overcome CRC chemoresistance.

Histone methylation modification is an important process involved in the regulation of gene expression, and this modification is controlled by histone methyltransferases and demethylases. In general, histone H3 lysine 27 trimethylation (H3K27me3) leads to gene repression, whereas histone H3 lysine 4 trimethylation (H3K4me3) and H3 lysine 36 methylation (H3K36me2/me3) promote gene transcription (Goldberg et al., 2007; Mikkelsen et al., 2007). It has been shown that abnormal histone methylation is closely related to tumor development (Greer and Shi, 2012). LSD1, a demethylase for H3K4, promotes stemness and chemoresistance in liver cancer cells by upregulating the Wnt/β-catenin signaling pathway (Lei et al., 2015), while it coordinates with the SIN3A/HDAC complex to maintain the sensitivity to chemotherapy in breast cancer (Yang et al., 2018). The H3K27me3 marker is acquired by the histone methyltransferase enhancer of zeste homolog 2 (EZH2) (Cao et al.,2002) and is removed by two demethylases KDM6A (UTX) and KDM6B (JMJD3) belonging to the Jumonji family (Agger et al., 2007; Xiang et al., 2007). It has been reported that EZH2 promotes chemotherapy resistance by inhibiting Schlafen family member 11 (SLFN11), expression in small cell lung cancer (Gardner et al., 2017). Interestingly, in B-cell lymphomas, inhibition of KDM6B activity by the small molecule inhibitor GSK-J4 promotes the efficacy of chemotherapeutic drugs (Mathur et al., 2017). This evidence suggests that H3K27me3 may play a different role in regulating drug resistance in different cancers and that the signaling pathways and their downstream target genes involved may be also different.

Here, we demonstrated that low H3K27me3 levels could result in CRC resistance to chemotherapeutics and predict poorer prognoses in patients with CRC. Downregulation of H3K27me3 using the small molecule inhibitor EPZ-6438 significantly promoted CRC cell resistance to oxaliplatin. Conversely, inhibition of the activity of demethylase KDM6A/KMD6B by GSK-J4 sensitized CRC to the effect of chemotherapeutic drugs by upregulating H3K27me3 levels *in vivo* and *in vitro*. Additionally, the effect of H3K27me3 modification on chemoresistance is achieved by targeting NOTCH2 expression, thus modulating the expression of stemness-related genes in CRC cells.

## Results

### Low H3K27me3 levels correlate with poor prognosis and enhanced oxaliplatin resistance in CRC

**Figure 1:**
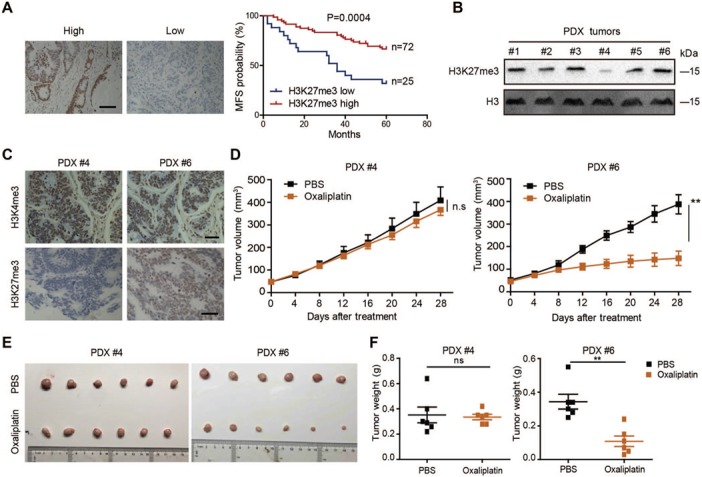
Decreased H3K27me3 levels are associated with poor prognosis and chemoresistance *in vivo*. (**A**) Kaplan–Meier plot of overall survival of patients based on H2K27me3 levels. A log-rank test was used for statistical analysis. Scale bar, 100 μm. MFS, metastasis-free survival. (**B**) Immunoblotting analysis of H3K27me3 in six PDX tumors. (**C**) Immunohistochemical analysis of H3K27me3 levels in two PDX samples. (**D–F**) PDX#4 and #6 tumors were subcutaneously injected into the nude mice. The mice were treated with oxaliplatin when the tumor volume reached 50 ± 10 mm^3^. Oxaliplatin was administered by i.p. injection (1 mg/kg) every 4 days for 28 days. Tumor growth curves (**D**), images of isolated tumors (**E**), and tumor weights (**F**) of each indicated group are shown (*n* = 6).

To explore the role of H3K27me3 in CRC, we analyzed H3K27me3 levels in 97 archived metastasis-free human CRC specimens by immunohistochemistry (IHC). Our results demonstrated that patients with low H3K27me3 level had shorter metastasis-free survival (MFS) times than those with high levels of H3K27me3, which indicated that H3K27 trimethylation might be an independent predictor of stratified risk for MFS (Figure 1A; Supplementary Table S1). Next, we detected the levels of H3K27me3 in six CRC patient-derived xenograft (PDX) samples. Western blot analysis indicated that H3K27me3 level varied dramatically in these PDX samples. H3K27me3 level in PDX#4 was significantly lower than that in the other samples (Figure 1B). In contrast, the expression of demethylase KDM6A/KDM6B in PDX#4 was significantly higher than that in other samples, while the expression of KDM6A/KDM6B in PDX#6 was relatively low (Supplementary Figure S1). Next, we performed an IHC assay to confirm the levels of H3K27me3 level in PDX#4 and PDX#6. Consistently, H3K27me3 level was higher in PDX#6 than PDX#4, while no significant difference was found in H3K4me3 level in these PDX samples (Figure 1C). Because H3K27me3 modification predicts a poor prognosis, we hypothesized that there might be a relationship between drug sensitivity and H3K27me3 level in CRC. To investigate the drug sensitivity of PDX#4 and PDX#6 *in vivo*, 1 mm^3^ tumor sections from PDX#4 and #6 were subcutaneously implanted into nude mice separately. When the tumor size reached 50 ± 10 mm^3^, we treated the mice with oxaliplatin every 4 days for 4 weeks. The mice bearing tumors from PDX#6 showed increased sensitivity to oxaliplatin than those bearing tumors from PDX#4. The tumor growth rates and weights were significantly inhibited by oxaliplatin treatment in mice bearing tumors from PDX#6, which had high levels of H3K27me3. Likewise, tumors with low H3K27me3 levels (tumors from PDX#4) were slightly inhibited by oxaliplatin (Figure 1D–F). These results indicated that low levels of H3K27me3 might be associated with oxaliplatin resistance.

### H3K27 demethylases KDM6A and KDM6B are required for oxaliplatin-based chemoresistance

**Figure 2:**
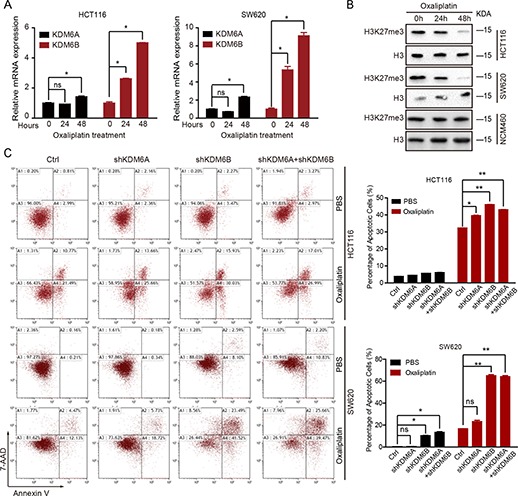
KDM6 is induced by oxaliplatin and inhibits oxaliplatin-induced apoptosis. (**A**) The KDM6A and KDM6B expression levels in HCT116 and SW620 cells treated with oxaliplatin (50 μM) for 24 or 48 h. ^*^P < 0.05 and ^**^P < 0.01 compared with control. (**B**) Immunoblotting analysis of H3K27me3 in HCT116, SW620, and NCM460 cells treated with oxaliplatin (50 μM) for 24 or 48 h. (**C**) HCT116 and SW620 cells depleted of KDM6A/KDM6B were exposed to oxaliplatin (50 μM) for 48 h. The percentage of cells entering apoptosis was determined by flow cytometry using APC-labeled Annexin V and 7-AAD staining.

Because of an association between low H3K27me3 level and oxaliplatin resistance, we questioned whether oxaliplatin treatment could induce H3K27 demethylation. First, we assessed the expression of two demethylases, KDM6A and KDM6B, after oxaliplatin treatment in CRC cell lines. Quantitative real-time reverse transcription PCR (qRT-PCR) assay showed a remarkable increase in the mRNA level of KDM6A and KDM6B in HCT116 and SW620 cell lines after oxaliplatin treatment for 24 and 48 h (Figure 2A). However, the mRNA level of EZH2 did not change (Supplementary Figure S2A). Meanwhile, there was an obvious reduction in H3K27me3 level after oxaliplatin stimulation in HCT116 and SW620 cell lines, while in normal colorectal epithelial cell line NCM460, oxaliplatin-induced reduction in H3K27me3 level was not observed (Figure 2B). Taken together, these results indicate that H3K27 demethylases KDM6A and KDM6B were involved in the regulation of chemoresistance in CRC cells.

Due to the induction of KDM6A and KDM6B by oxaliplatin, we generated KDM6A- and KDM6B-depleted HCT116 and SW620 cells using an shRNA knockdown system (Supplementary Figure S2B and Table S3). Oxaliplatin treatment in KDM6A/KDM6B knockdown cells significantly increased the percentage of apoptotic cells compared with that in control cells, and KDM6B played a major role in this process (Figure 2C).

### Small molecule inhibitors GSK-J4 and EPZ-6438 modulate oxaliplatin-induced apoptosis in CRC cells

**Figure 3:**
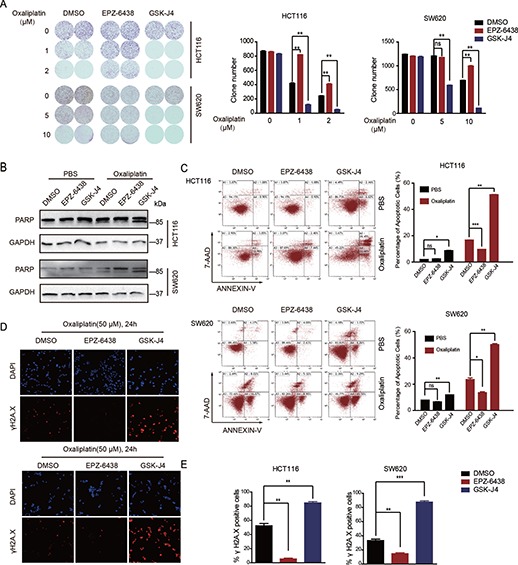
Shifts in H3K27me3 regulate oxaliplatin-induced apoptosis in CRC cells. (**A**) HCT116 and SW620 cells were treated with GSK-J4 (1 μM) or EPZ-6438 (10 μM) and exposed to oxaliplatin for 7 days. Clonogenic survival assays were performed. (**B**) Immunoblotting analysis of PARP in HCT116 and SW620 cells treated with GSK-J4 (1 μM) or EPZ-6438 (10 μM) and exposed to oxaliplatin for 48 h. (**C**) HCT116 and SW620 cells were treated with GSK-J4 (1 μM) or EPZ-6438 (10 μM) and exposed to oxaliplatin (50 μM) for 48 h. The percentage of cells entering apoptosis was determined by flow cytometry using APC-labeled Annexin V and 7-AAD staining. (**D**) Immunofluorescence staining of γH2A.X formation (red fluorescence) in the indicated cells treated with oxaliplatin (50 μM) for 24 h. The nuclei were stained with DAPI (blue fluorescence). (**E**) Quantification of γH2A.X staining in **D**. The number of positive cells was averagely estimated in three fields of each section.

Next, we selected two inhibitory molecules that target the enzymes involved in the modification of H3K27 methylation process to further investigate the function of H3K27me3 modification in oxaliplatin-induced apoptosis. GSK-J4 is an inhibitor of KDM6A/KDM6B (Kruidenier et al., 2012), and EPZ-6438 inhibits the activity of the methyltransferase EZH2 (Knutson et al., 2013). First, we tested the effect of these two inhibitors on H3K27me3 level in CRC cell lines. As shown in Supplementary Figure S3A, EPZ-6438 treatment significantly decreased the H3K27me3 level, while GSK-J4 had the opposite effect. Then we performed colony formation assays to investigate the effect of GSK-J4 and EPZ-6438 on cell survival upon oxaliplatin stimulation. Our data revealed that oxaliplatin treatment decreased the number of colonies in HCT116 and SW620 cell lines. When we stimulated CRC cells with EPZ-6438 and oxaliplatin, more cells survived to form colonies. However, when we combined GSK-J4 and oxaliplatin, the number of CRC colonies decreased dramatically (Figure 3A). To understand the mechanism, we performed a flow cytometry assay and found that neither EPZ-6438 nor GSK-J4 influenced the cell cycle distribution (Supplementary Figure S3B). Furthermore, treatment of HCT116 cells with GSK-J4 at a concentration of 1 μM did not affect the expression of the apoptosis-related genes BCL-2, BAX, and BCL-xl (Supplementary Figure S3C). Interestingly, we observed an increase in cleaved poly ADP-ribose polymerase (PARP) expression, which is an inactive form of PARP disrupting DNA repair and resulting in apoptosis (Herceg and Wang, 2001), in GSK-J4-treated CRC cells after oxaliplatin stimulation, while EPZ-6438 inhibited this process (Figure 3B). Moreover, the flow cytometry assay revealed that GSK-J4 increased oxaliplatin-induced apoptosis, and EPZ-6438 decreased the percentage of apoptotic cells (Figure 3C). As a marker of platinum drug-induced apoptosis (Pilch et al., 2003), γH2A.X foci formation was assessed in oxaliplatin-treated SW620 and HCT116 cells. As indicated in Figure 3E, the addition of EPZ-6438 induced weak γH2A.X signals, whereas GSK-J4 treatment increased γH2A.X fluorescence (Figure 3D and E). Collectively, these results demonstrated that the inhibitor GSK-J4 enhanced oxaliplatin-induced apoptosis via the upregulation of H3K27me3 level, while EPZ-6438 reduced apoptosis by downregulating H3K27me3 level *in vitro*.

### GSK-J4 improves drug sensitivity in oxaliplatin-resistant PDX tumors

**Figure 4:**
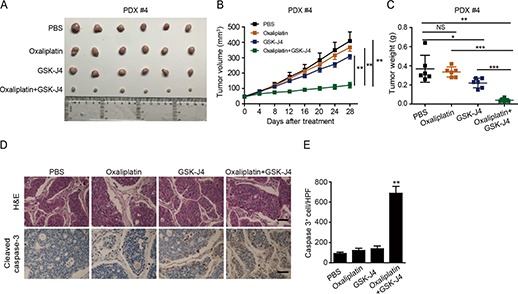
Increased H3K27me3 levels enhance oxaliplatin efficacy in chemoresistant PDXs. (**A**–**C**) PDX#4 tumors were subcutaneously injected into nude mice. The mice were treated with oxaliplatin when the tumor volume reached 50 ± 10 mm^3^. Oxaliplatin was administered by i.p. injection (1 mg/kg) every 4 days for 28 days and GSK-J4 was administered by i.p. injection (100 mg/kg) for 20 consecutive days. Images of isolated tumors (**A**), tumor growth curves (**B**), and tumor weights (**C**) of the indicated group are shown. (**D**) PDX tumors stained with hematoxylin-eosin (H&E) or cleaved caspase 3 antibody. Apoptotic cells were visualized. Scale bar, 100 μm. (**E**) Quantification of cleaved caspase 3 levels in PDX tumors.

Our data indicated that GSK-J4 treatment overcame oxaliplatin resistance in CRC cells by upregulating H3K27me3 level; therefore, we assessed the response of oxaliplatin-resistant PDX tumors *in vivo* to GSK-J4 administration. Tumors (1 mm^3^ sections) from PDX#4 were subcutaneously inoculated into BALB/C nude mice. We subsequently observed tumor growth in the mice following intraperitoneal (i.p.) injection of GSK-J4 combined with oxaliplatin. The results demonstrated that the combination of GSK-J4 and oxaliplatin significantly inhibited the tumor growth rate and weight compared with the group that was treated only with oxaliplatin (Figure 4A–C). Moreover, the body weight did not change under the conditions of treatment (Supplementary Figure S4). Furthermore, tumors receiving the combination treatment of oxaliplatin and GSK-J4 displayed more cleaved caspase 3 expression compared with those treated with oxaliplatin or GSK-J4 alone as determined by IHC (Figure 4D and E). Taken together, GSK-J4 could be used as a potential clinical candidate to improve oxaliplatin sensitivity in chemoresistant CRC patients with low levels of H3K27me3.

### GSK-J4 inhibits the oxaliplatin-induced NOTCH signaling pathway

To explore the mechanism of how H3K27me3 modulates CRC chemoresistance, we performed RNAseq analysis using the GSK-J4- or oxaliplatin-treated HCT116 cells.

**Figure 5:**
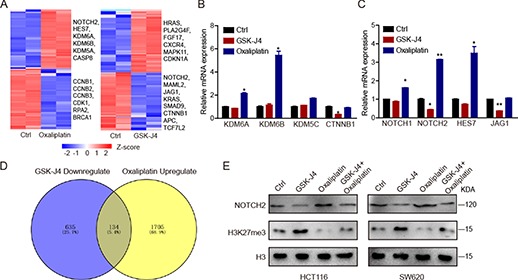
GSK-J4 inhibits oxaliplatin-induced NOTCH2 expression. (**A**) Heatmap shows the expression profiles of variably expressed genes across control, oxaliplatin (50 μM)-treated, and GSK-J4 (1 μM)-treated cells. (**B** and **C**) Detection of GSK-J4 (1 μM) or oxaliplatin (50 μM)-induced changes in KDM genes (**B**) or NOTCH-related genes (**C**). (**D**) Venn diagram indicates oxaliplatin-upregulated and GSK-J4-downregulated genes. (**E**) Western blot detection of NOTCH2 expression after oxaliplatin (50 μM) and GSK-J4 (1 μM) treatment.

Oxaliplatin treatment resulted in the upregulation of 1839 genes and the downregulation of 1913 genes. Consistently, oxaliplatin significantly increased the expression of KDM6A and KDM6B. In addition, after GSK-J4 stimulation, 728 genes were upregulated, and 769 genes were downregulated (Figure 5A). A qRT-PCR assay was performed to verify the mRNA expression of KDM6A, KDM6B, KDM5C, and CTNNB1 (Figure 5B). Interestingly, there was an overlap of 134 genes between the GSK-J4 downregulated genes and the upregulated genes by oxaliplatin, and thus, these genes might be required for GSK-J4-enhanced oxaliplatin-based chemosensitivity (Figure 5D). Previous studies have shown that NOTCH signaling can be activated by receptor tyrosine kinases inhibitors (Liau et al., 2017); therefore, we evaluated the expression of NOTCH-related genes after oxaliplatin treatment. We observed that NOTCH1, NOTCH2, and HES7 genes were upregulated by oxaliplatin. However, only the expression of NOTCH2 was suppressed by GSK-J4 (Figure 5C and D). Western blot assay confirmed that oxaliplatin significantly increased NOTCH2 expression, while the addition of GSK-J4 inhibited the upregulation of NOTCH2 (Figure 5E). Taken together, these data suggest that GSK-J4 sensitized CRC cells to oxaliplatin by regulating NOTCH signaling.

### GSK-J4 inhibits NOTCH2 expression by enhancing the trimethylation of H3K27 at the NOCTH2 loci

**Figure 6:**
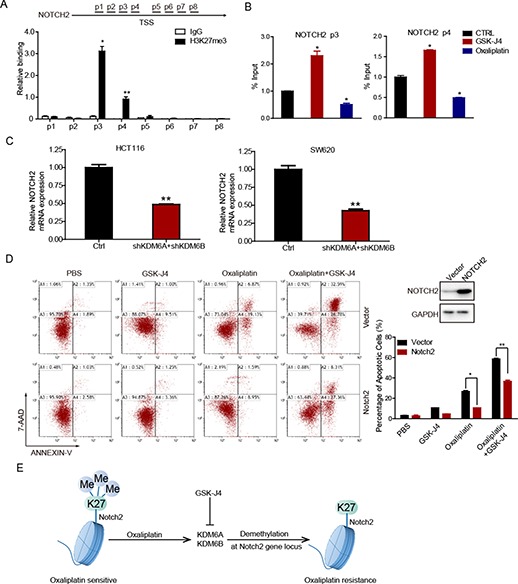
Oxaliplatin and GSK-J4 alter NOTCH2 expression by affecting the level of H3K27me3 transcription initiation region. (**A**) ChIP-qPCR analysis of H3K27me3 binding to the NOTCH2 promoter in HCT116 cells. (**B**) ChIP-qPCR analysis of anti-H3K27me3 in control and GSK-J4 (1 μM) or oxaliplatin (50 μM)-treated cells. (**C**) qRT-PCR detection of KDM6A/KDM6B expression after NOTCH2 knockdown. (**D**) Western blot detection of NOTCH2 expression in NOTCH2 overexpression HCT116 cells (upper right panel). NOTCH2 overexpression and control HCT116 cells were treated with GSK-J4 (1 μM) and exposed to oxaliplatin (50 μM) for 48 h. The percentage of cells entering apoptosis was determined by flow cytometry using APC-labeled Annexin V and 7-AAD staining. (**E**) Graphic illustration of the relationship between H3K27me3 and oxaliplatin resistance.

To investigate how GSK-J4 regulated NOTCH2 expression, we performed a chromatin immunoprecipitation (ChIP) assay. The distribution of H3K27me3 was mainly at the sites 1 kb upstream of the NOTCH2 transcription start site (TSS) (p3) and 500 bp upstream of the NOTCH2 TSS (p4) (Figure 6A). As an inhibitor of KDM6A/KDM6B, GSK-J4 was shown to upregulate H3K27me3 level. Therefore, we further compared H3K27me3 modifications in the above two regions after GSK-J4 or oxaliplatin stimulation. GSK-J4 treatment significantly increased the level of repressive histone marker H3K27me3 at sites p3 and p4 compared with that in control cells. However, oxaliplatin stimulation obviously eliminated H3K27me3 modification levels in these regions (p3, p4) of the NOTCH2 gene (Figure 6B), which may be the results of high KDM6A and KDM6B levels induced by oxaliplatin (Figure 2A). In order to identify the underlying mechanisms, we analyzed the expression levels of NOTCH2 in the parental and KDM6A and KDM6B knockdown CRC cells by qRT-PCR; the results indicated that knockdown of KDM6A and KDM6B downregulated the NOTCH2 expression (Figure 6C). At the same time, we also found that the overexpression of NOTCH2 can effectively inhibit the apoptosis caused by co-treatment of oxaliplatin and GSK-J4 (Figure 6D). In summary, our data revealed that oxaliplatin induces NOTCH2 expression by upregulating KDM6A/KDM6B, resulting in drug resistance in CRC cells. GSK-J4, an inhibitor of both KDM6A and KDM6B, effectively enhanced H3K27me3 modification at NOTCH2 loci and sensitized CRC cells to oxaliplatin therapy (Figure 6E).

### H3K27me3 modification is required for CSC maintenance

Colon CSCs are thought to be an important cause of chemotherapy resistance (Dean et al., 2005; O’Brien et al., 2007; Maugeri-Sacca et al., 2011), and NOTCH signaling plays a crucial role in the maintenance of CSCs (Pannuti et al., 2010; Long et al., 2018).

**Figure 7:**
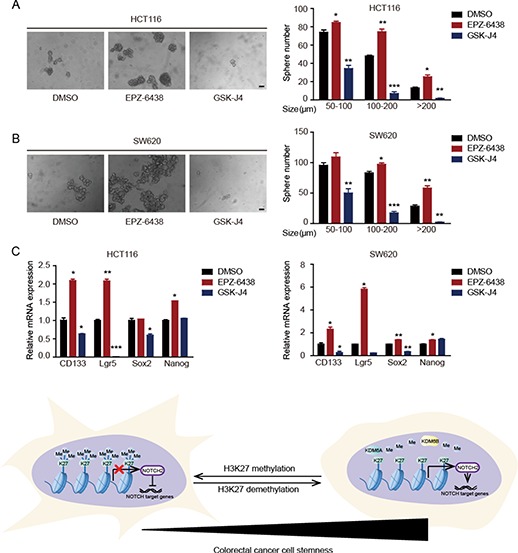
H3K27me3 level regulates the stemness of colorectal CSCs. (**A** and **B**) Oncosphere formation in GSK-J4 (1 μM) or EPZ-6438 (10 μM)-treated HCT116 (**A**) and SW620 (**B**) cells. The number and size of spheres derived from GSK-J4- or EPZ-6438-treated cells are compared with those of control cells. (**C**) qRT-PCR analysis of the expression of stemness genes in control and GSK-J4 (1 μM) or EPZ-6438 (10 μM)-treated cells. (**D**) A graphic illustration of findings in this study.

Since we have demonstrated that H3K27me3 regulates the expression of NOTCH2 and modulates the sensitivity of CRC cells to the chemotherapeutic drug oxaliplatin, we hypothesized that the effect of H3K27me3 modification on drug sensitivity was likely due to its regulation on colorectal CSCs. To characterize the function of H3K27me3 modification in colorectal CSCs, two inhibitors, EPZ-6438 and GSK-J4, were used in an oncosphere assay. The addition of GSK-J4 inhibited oncosphere formation, while EPZ-6438 significantly enhanced this process (Figure 7A and B). Consistent with the oncosphere formation results, stemness-related genes, such as CD133, Lgr5, NANOG, and Sox2, were upregulated by EPZ-6438 treatment but downregulated by GSK-J4 (Figure 7C). Taken together, these data demonstrate that H3K27me3 plays a critical role in the maintenance of colorectal CSCs and, therefore, in regulating chemoresistance in CRC cells (Figure 7D).

## Discussion

In stem cells, the degree of H3K27me3 is usually low in the promoter region of stem genes. As the degree of H3K27me3 in these promoter regions increases, stem cells differentiate into different types of cells (Conway et al., 2015). Clinical studies have shown that poorly differentiated CRC is closely associated with shorter survival and worse prognosis (Yoshida et al., 2011). Here, we report for the first time that a low level of H3K27me3 predicts a poor prognosis in CRC patients. We demonstrated that low H3K27me3 levels maintained the stemness of colorectal CSCs and thus enhanced the resistance of CRC cells to chemotherapeutic drugs. First, oxaliplatin-induced apoptosis was significantly increased after the elevation of H3K27me3 levels by GSK-J4 treatment in CRC cells *in vitro*. Next, the combination of GSK-J4 and oxaliplatin significantly inhibited tumor growth compared with that in the group treated only with oxaliplatin in an oxaliplatin-resistant PDX model. Moreover, GSK-J4 remarkably inhibited oncosphere formation and the expression of stemness-related genes. These findings suggest that H3K27me3 might be a potential target for overcoming chemoresistance in CRC.

In this article, we found that overexpression of NOTCH2 does not completely reduce the apoptotic proportion induced by the combination of GSK-J4 and oxaliplatin to the same level as oxaliplatin treatment alone. There might be other genes involved in the process. Our transcriptome sequencing (RNA-seq) analysis showed that GSK-J4 treatment significantly downregulate other stem cell-related genes such as CTNNB1, TEAD4, and TNSK2. However, ChIP assay demonstrated that H3K27me3 cannot be enriched at the promoter regions of these genes (data not shown), which indicated that these genes are not directly regulated by the modification of H3K27me3 on their promoter region. It has been reported that UTX/JMJD3 can enhance the WNT3 gene expression by removing H3K27me3 from its gene promoter, thus promoting β-catenin-mediated gene expression in the process of endoderm differentiation (Jiang et al., 2013). These results indicated that other mechanisms may participate in the process that GSK-J4 treatment overcome oxaliplatin resistance in CRC.

The effect of H3K27me3 levels on chemoresistance remains controversial in different types of cancers. It has been reported that low EZH2 expression, which reduces H3K27 methylation, promoted drug resistance in BRCA-2-deficient breast cancer cells by the regulation of genomic stability (Rondinelli et al., 2017). EPZ-6438, also named tazemetostat, an inhibitor of EZH2, has entered phase II clinical trials for non-Hodgkin’s lymphoma treatment (Morschhauser et al., 2016). We found that EPZ-6438 significantly enhanced oxaliplatin-based resistance, but it had little effect on CRC proliferation. Moreover, some studies have suggested that the upregulation of H3K27me3 by GSK-J4 promoted the sensitivity of cancer cells to chemotherapeutic drugs in lung cancer and glioblastoma (Dalvi et al., 2017; Liau et al., 2017; Mathur et al., 2017). Moreover, GSK-J4 treatment sensitized diffuse large B-cell lymphoma to chemotherapy drugs through the downregulation of B-cell receptor signaling and Bcl-6 (Mathur et al., 2017). However, inhibition of EZH2 expression or its methyltransferase activity promoted chemosensitivity through the induction of SLFN11 expression in small cell lung cancer (Gardner et al., 2017), which is contrary to our results in CRC. Taken together, the modification of H3K27me3 plays opposite roles in various cancer types, and the mechanisms underlying remains different. Moreover, we first demonstrated that upregulation of H3K27me3 by GSK-J4 promoted the sensitivity of CRC cells to oxaliplatin treatment.

In osteoblast, low levels of H3K27me3 promote the expression of Bcl-2 to inhibit apoptosis (Yang et al., 2016). In our experiment, we found that the combination of GSK-J4 and oxaliplatin caused two times more apoptotic cells than GSK-J4 or oxaliplatin treatment separately *in vitro* (Figure 3D) and *in vivo* (Figure 4E). We suspected that although GSK-J4 treatment inhibits the expression of NOTCH2 and induced apoptosis, the apoptosis is not the result of inactivation of NOTCH signaling. When implicating with oxaliplatin, the activated NOTCH signaling induces the expression of downstream stem cell-related genes and promote the transport of drugs (Liu et al., 2013; Pannuti et al., 2010), thus the degree of apoptosis is enhanced.

Although our results *in vitro* and *in vivo* indicate that higher H3K27me3 levels could sensitize CRC to oxaliplatin, the clue is based on a single case of H3K27me3 low PDX (#4). More PDX are still needed to verify the conclusion that we draw. At the same time, Chen et al. (2016b) also reported that EZH2 promoted CRC stem-like cell expansion, which turned out contrary to ours. It is possible that EZH2 has a functional mode that independently of histone methyltransferase activity (Yan et al., 2013). Although there are articles reporting that EZH2 increases drug resistance, there are also articles showing that inhibition of EZH2 activity can promote drug sensitivity (Gollner et al., 2017; Rondinelli et al., 2017).

EZH2 is considered to be an oncogene, but inhibition of EZH2 activity in some solid tumors does not inhibit tumor growth due to changes in H3K27 trimethylation caused by EZH2 and also causes changes in H3K27 acetylation (Huang et al., 2018). In our experiment, we also found that the use of EPZ-6438 to inhibit EZH2 activity in CRC does not inhibit tumor growth.

In summary, we found that low levels of H3K27me3 were associated with poor prognosis, and increasing H3K27me3 levels sensitized CRC cells to oxaliplatin *in vitro*. The combination of GSK-J4 and oxaliplatin predominantly inhibited tumor growth in the oxaliplatin-resistant PDX model. GSK-J4 induced H3K27me3 level and significantly inhibited the self-renewal capability of colorectal CSCs through the downregulation of NOTCH2 expression. Because a crucial role is played by H3K27me3 modification in regulating resistance to oxaliplatin, elevating its expression by GSK-J4 may be an effective strategy to overcome chemoresistance in CRC treatment.

## Materials and methods

### Animal experiments

Four-week-old nude mice were purchased from Shanghai Laboratory Animal Center, Chinese Academy of Sciences. All experiments were performed under the guidelines for the care and use of laboratory animals. PDX models were established according to previously published reports (Choi et al., 2016). The nude mice were randomly divided into groups, then PDX tumors were cut to 1 mm^3^ in size and implanted subcutaneously into the flanks of the nude mice. Drug treatment was initiated when the tumor volume reached 50 ± 10 mm^3^. Oxaliplatin was administered by i.p. injection (1 mg/kg) every 4 days for 28 days, and GSK-J4 was administered via i.p. injection (100 mg/kg) for 20 consecutive days. After 4 weeks, the mice were sacrificed, and the tumors were dissected. The tumor weight was measured, and the tumor volume was calculated using the following formula: A × B^2^ × 0.5, with A representing the longest diameter and B the shortest diameter.

### Immunohistochemical analyses

IHC analyses were performed as described previously (Chen et al., 2016a) using an anti-H3K27me3 antibody (1:200 dilution) and a cleaved caspase 3 antibody (1:1000 dilution). Biospecimens, confirmed by two independent pathologists, were collected from patients with CRC undergoing surgical resection. Fundamentally, the quantification method was based on a multiplicative index of staining proportion and staining extent in the cores. Scores ≥2 points were considered high levels of H3K27me3, while scores <2 were considered low levels of H3K27me3.

### Cell culture reagents and antibodies

Human CRC cell lines HCT116 and SW620 were purchased from American Type Culture Collection. Cells were cultured in Dulbecco’s modified Eagle medium containing 10% fetal calf serum medium. All CRC cells were negative for mycoplasma contamination prior to use.

Oxaliplatin, GSK-J4, and EPZ-6438 were purchased from Selleck. DAPI (#5748) was obtained from R&D Systems. The following antibodies were used: H3K27me3 (9733, Cell Signaling Technology) for IHC, H3K27me3 (07–449; Millipore) for ChIP and western blot, H3K4me3 (61379, ActiveMotif), H3 (sc-10809, Santa Cruz), PAPR (9542, Cell Signaling Technology), γH2A.X (3322, Abways), and cleaved caspase 3 (9664, Cell Signaling Technology).

### RNA extraction and real-time PCR

Total RNA was isolated using TRIzol reagent (TaKaRa) according to the manufacturer’s protocol. To obtain cDNA, PrimeScript™ RT Master Mix (TaKaRa, RR036A) was used to generate cDNA following the manufacturer’s instructions using 1.5 μg RNA as the template. GAPDH was used as an internal control gene. The qRT-PCR reagents were purchased from TaKaRa, and a 7500 Fast Real-Time PCR System (Applied Biosystems) was used for qRT-PCR. All samples were analyzed in triplicate. The error bars represent the standard error of mean, and statistical significance was calculated using one-tailed, unpaired *t*-test. Primer sequences are presented in Supplementary Table S2.

### Western blot analysis

The harvested cells were washed twice with phosphate buffer saline (PBS), dissolved in loading buffer, and lysed at 100°C for 10 min. Proteins were separated by SDS-PAGE gel and transferred onto a polyvinylidene fluoride (PVDF) membrane (Immobilon P, Millipore). The membrane was blocked in 10% nonfat milk for 30 min followed by immunoblotting with primary and HRP-labeled secondary antibodies.

### Flow cytometry

A total of 1 × 10^5^ cells were plated in 6-well plates for 48 h. After trypsin digestion and washing with PBS, APC-Annexin V (Invitrogen) and 7-AAD (BD Biosciences) were added for 30 min at room temperature in the dark, prior to flow cytometry analysis. The data were analyzed with Kaluza analysis software (Kaluza Analysis 1.3, Beckman Coulter).

### Colony formation and oncosphere formation assays

In the colony formation assays, cells were cultured continuously in a 10% serum medium for 7 days, then the medium was removed, and the cells were stained with bromophenol blue for 20 min. For sphere formation assays, the cells were suspended in serum-free medium and plated in low-attachment 24-well plates. The serum-free medium contained DMEM/F-12 supplemented with B27 (1:50) supplement, EGF (20 ng/ml), and FGF (10 ng/ml). Seven days after continuous cultivation, the number of spheres was recorded under a microscope.

### RNA-Seq

The total mRNA was obtained from HCT116 cells treated with either oxaliplatin or GSK-J4 for 48 h. After total RNA extraction, samples were processed by Hi-Seq 2500 by OE Biotech. Gene expression levels were analyzed by the software HTSeq-count and Cufflinks (version 0.6.1) (Roberts et al., 2011; Anders et al., 2015). All differentially expressed genes generated by the software DESeq (version 1.34.1) were then analyzed by the DAVID bioinformatics platform to enrich biological terms.

### ChIP

HCT116 cells were treated with oxaliplatin or GSK-J4 for 48 h. Then 1 × 10^6^ cells were fixed with formaldehyde for 10 min and sonicated with UCD-300 (Bioruptor). Incubation with antibodies was reversed by cross-linking using the EZ-ChIP immunoprecipitation kit (17–371; Millipore). The antibody used for ChIP was polyclonal rabbit anti-H3K27me3 (07–449; Millipore). Primer sequences (5′–3′) used for PCR amplification are listed in Supplementary Table S2.

### Statistical analyses

All experiments were performed using three independent replicates from cells. Unless otherwise stated, the data in the graph are presented as mean ± SD, and the statistical significance was evaluated using a two-tailed Student’s *t*-test. Independent prognostic factors were determined by Cox proportional hazard regression models. Differences reached significance at *P* < 0.01 (**) and *P* < 0.05 (*).

## Supplementary Material

Supplementary_material_mjz032
