# Supplementary material for: Elevating H3K27me3 level sensitizes colorectal cancer to oxaliplatin
Source: J Mol Cell Biol. 2019 May 8;12(2):125–37. doi: 10.1093/jmcb/mjz032 (PMC7109602; doi:10.1093/jmcb/mjz032)
Supplement: Supplementary_material_mjz032 [file supplementary_material_mjz032.pdf]

Supplementary Figures

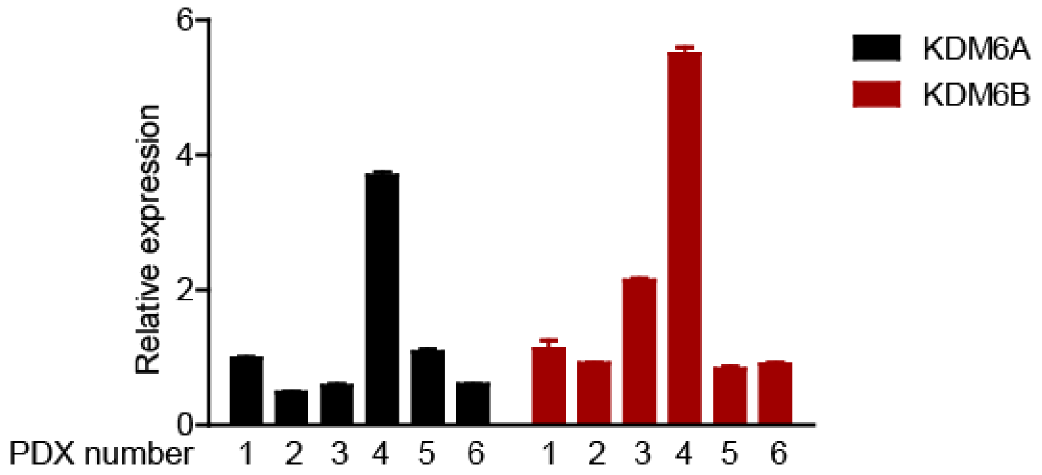

Supplementary Figure S1. q-PCR analysis of KDM6A and KDM6B expression in six PDX tumors.

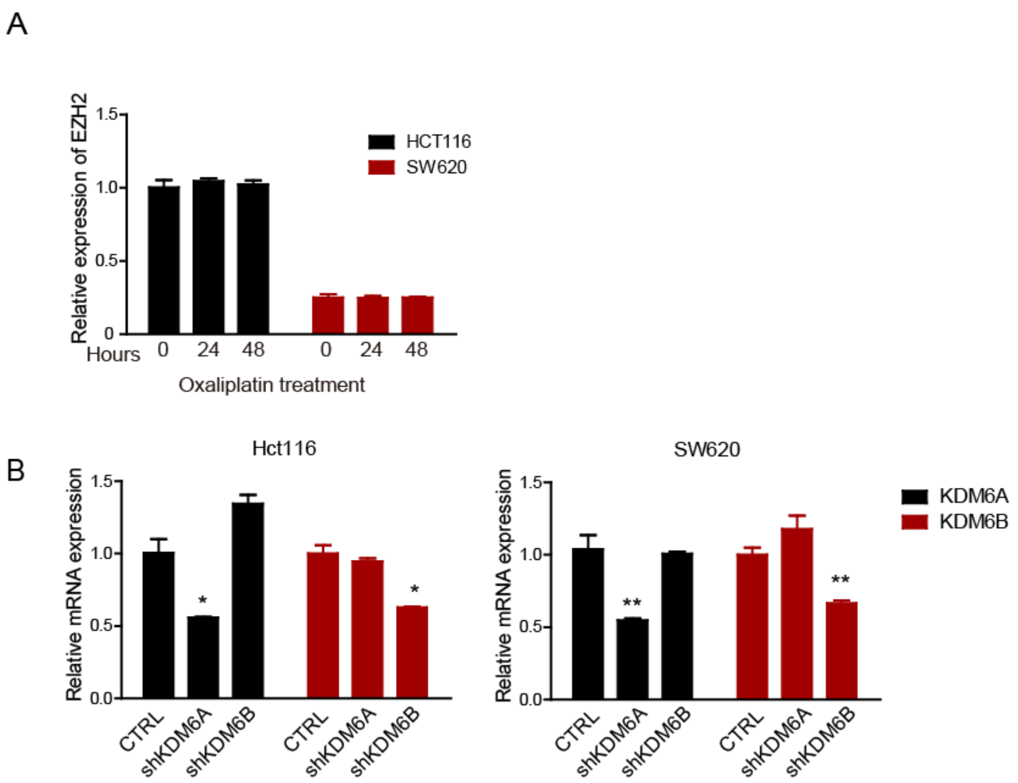

Supplementary Figure S2. (A) The EZH2 expression levels in HCT116 cells and SW620 cells treated with oxaliplatin (50 μM)for 24 or 48 hours.(B)q-PCR analysis of KDM6A and KDM6B expression in control and KDM6A/KDM6B knockdown cells.

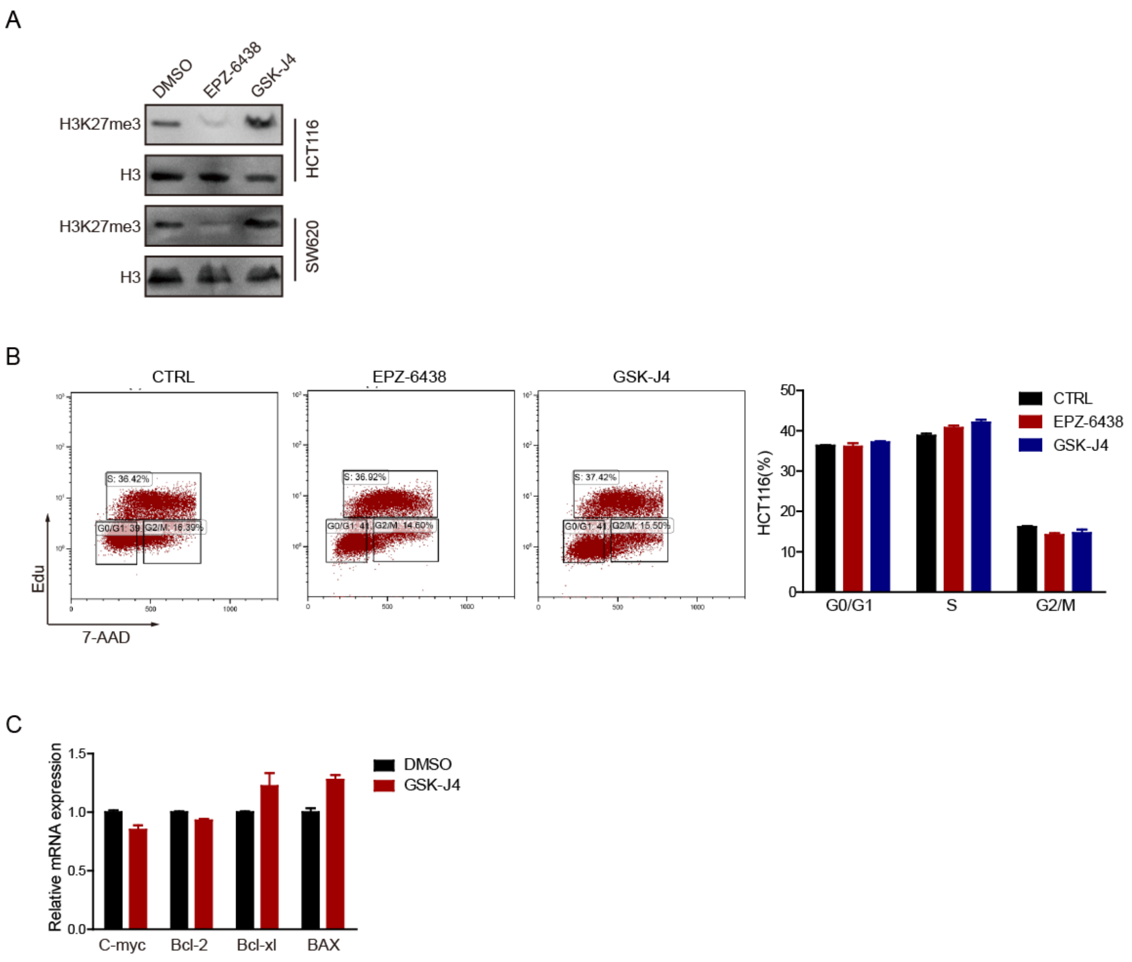

Supplementary Figure S3. (A) Immunoblotting analysis of the H3K27me level in HCT116 cells and SW620 cells treated with GSK-J4 (1 μM)and EPZ-6438 (10 μM)at 48 hours.(B) Flow cytometry was used to evaluate the effects of GSK-J4 or EPZ-6438 treatment on cell-cycle arrest in HCT116 . (C) The C-myc, BAX, BCL2 , BCL-xl expression levels in HCT116 cells treated with GSK-J4(1μM) for 48 hours. *P<0.05 and **P<0.01 compared with control.

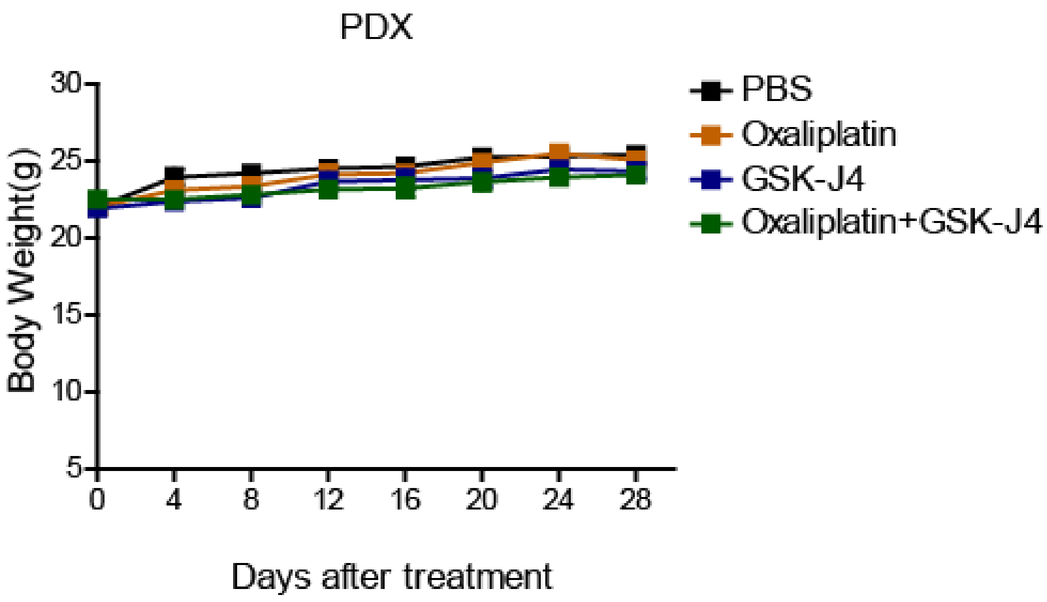

Supplementary Figure S4. The body weights of the PDX mice were measured and recorded for each group throughout the experiment (n =6).

Supplementary Tables

Table S1. Tissue microarray data and clinical information were used for analyses												
**NO**	**operation time**	**suvival state**	**survival time(months)**	**gender**	**age**	**metastas**	**pathological grading**	**Tumor size**	**T**	**N**	**M**	**The pathological grade**
1	2007.6	death	32	female	52	no	Ⅱ	5×2.5×1cm	T3	N1b	M0	0
2	2007.6	death	7	female	81	no	Ⅱ	4×3×1.5cm	T4a	N1b	M0	0
3	2007.6	survive	60	female	78	no	Ⅱ	6×5×1.5cm	T4a	N0	M0	1
4	2007.6	death	9	female	64	no	Ⅱ	4×3×1.5cm	T3	N0	M0	3
5	2007.7	death	76	female	54	no	Ⅱ	5×4×1cm	T3	N0	M0	3
4	2007.6	survive	60	female	74	no	Ⅲ	10×5×1.5cm	T3	N2a	M0	3
5	2007.6	survive	60	female	81	no	Ⅱ	4.5×4×1.5cm	T3	N0	M0	3
6	2007.7	death	2	female	79	no	Ⅱ	3.5×3×1cm	T2	N0	M0	3
7	2007.8	survive	60	male	74	no	Ⅲ	9×6×1.5cm	T3	N2a	M0	0
8	2007.7	survive	60	female	60	no	Ⅱ	3×2×1cm	T4b	N1a	M0	0
9	2007.8	death	8	female	60	no	Ⅱ	7×3×1.5cm	T3	N1a	M0	3
10	2007.9	survive	60	male	79	no	Ⅲ	6×4.5×1cm	T3	N1b	M0	2
11	2007.7	survive	60	female	54	no	Ⅱ	5×4×1cm	T4a	N0	M0	2
12	2007.8	death	43	female	50	no	Ⅱ	5×4×1cm	T3	N0	M0	2
13	2007.7	death	25	male	78	no	Ⅲ	4.5×3×1.5cm	T3	N1b	M0	2
14	2007.8	survive	60	female	70	no	Ⅱ	5×4×1cm	T3	N1b	M0	2
15	2007.9	death	39	female	62	no	Ⅱ	8×7×1cm	T3	N0	M0	3
16	2007.9	survive	60	female	66	no	Ⅱ	6×4.5×1.5cm	T3	N1a	M0	0
17	2007-10-18	death	16	female	65	no	Ⅱ	9×3×3cm	T3	N1b	M0	2
18	2007-10-26	survive	60	female	70	no	Ⅱ	5×4×1cm	T3	N0	M0	2

19	2007-12-11	death	13	female	71	no	II	4×3×1cm	T3	N1a	M0	3
20	2007-12-20	survive	60	female	78	no	II	4×2×0.6cm	T3	N0	M0	3
21	2007-12-11	death	12	female	61	no	II	5×4.5×2cm	T3	N0	M0	3
22	2008.3	survive	60	female	58	no	II	6×3×1cm	T3	N0	M0	3
23	2007-12-13	death	58	female	75	no	II	4×4×1cm	T4a	N0	M0	2
24	2007.9	survive	60	female	43	no	II	5×4.5×1.5cm	T3	N2a	M0	0
25	2008.1	death	60	female	76	no	II	6×4.5×1.5cm	T3	N0	M0	1
26	2007-12-5	survive	60	female	61	no	II	9×4.5×1.5cm	T3	N2b	M0	2
27	2008.3	death	18	female	72	no	II	7×5×2cm	T4a	N1a	M0	2
28	2008.3	death	60	female	61	no	II	5×4×1.5cm	T3	N1b	M0	3
29	2008.3	death	36	female	64	no	II	6×4×1.5cm	T3	N0	M0	1
30	2008.3	death	32	female	55	no	II	3×2.5×1.8cm	T1	N0	M0	2
31	2008.4	death	40	female	63	no	II	4×3.5×2.5cm	T3	N0	M0	0
32	2008.3	death	24	female	70	no	II	3.5×3.5×0.8cm	T3	N1b	M0	1
33	2007.6	survive	60	male	62	no	II	7×4×1.8cm	T4a	N0	M0	2
34	2007.9	survive	60	male	65	no	II	5×4×1cm	T3	N1a	M0	3
35	2007-10-22	survive	60	male	80	no	II	2.8×2.5×1cm	T4b	N0	M0	3
36	2007-11-20	survive	60	male	68	no	II	3.5×2.7×1cm	T3	N0	M0	3
37	2007-12-13	survive	60	male	65	no	II	10×7×1.5cm	T4a	N1b	M0	2
38	2007-12-17	survive	60	male	61	no	II	3.3×2.7×1cm	T2	N0	M0	3
39	2008.1	survive	60	male	68	no	II	6×3.5×1.5cm	T3	N0	M0	2
40	2008.3	survive	60	male	77	no	II	5×3.5×1cm	T3	N0	M0	3

41	2008.3	survive	60	male	62	no	Ⅱ	5×4.5×1.5cm	T3	N1a	M0	3
42	2008.3	survive	60	male	75	no	Ⅱ	5×5×1.5cm	T3	N2a	M0	0
43	2008.3	survive	60	male	58	no	Ⅱ	3×3×1cm	T3	N1a	M0	3
44	2008.3	survive	60	male	72	no	Ⅱ	6.5×5×1.5cm	T3	N0	M0	1
45	2008.3	survive	60	male	65	no	Ⅱ	6×5×1.5cm	T3	N0	M0	2
46	2008.3	survive	60	male	80	no	Ⅱ	2.5×2.5×1cm	T3	N0	M0	1
47	2007.6	survive	60	male	72	no	Ⅱ	4×3×1.5cm	T4a	N0	M0	2
48	2008.3	survive	60	male	71	no	Ⅱ	4.5×3×1.5cm	T3	N0	M0	2
49	2007.6	death	10	male	60	no	Ⅱ	3×2.5×1.5cm	T3	N0	M0	3
50	2007.7	death	91	male	65	no	Ⅱ	4×4×1.5cm	T3	N0	M0	3
51	2007.7	death	43	male	83	no	Ⅱ	6×4.5×1.5cm	T3	N2b	M0	3
52	2007-10-17	death	7	male	74	no	Ⅱ		T4b	N2a	M0	2
53	2007-10-26	death	5	male	75	no	Ⅱ	3×2×1.8cm	T3	N0	M0	2
54	2007-11-22	death	44	male	70	no	Ⅱ	6×3.5×1.5cm	T3	N1b	M0	3
55	2007-12-3	death	58	male	58	no	Ⅱ	4×4×1.5cm	T3	N0	M0	0
56	2008.1	death	16	male	74	no	Ⅱ	4×4×1cm	T3	N1a	M0	1
57	2008.3	death	38	male	60	no	Ⅱ	①4.5×3.5×1cm；②1.5×0.8×0.8cm	T3	N0	M0	1
58	2008.4	death	57	male	76	no	Ⅱ	2×1.5×1cm	T3	N1a	M0	2
59	2007.9	death	2	male	51	no	Ⅱ	3×3×1cm	T3	N0	M0	2
60	2008.1	death	36	male	69	no	Ⅱ	5×5×3cm	T3	N1b	M0	3

61	2008.1	death	51	male	67	no	Ⅱ	5×4×3cm	T4b	N2a	M1a	3
62	2008.1	death	4	male	73	no	Ⅱ	4×4×2cm	T4a	N0	M0	3
63	2007.6	survive	60	female	64	no	Ⅰ-Ⅱ	5×5×1cm	T4b	N0	M0	3
64	2007.7	survive	60	female	60	no	Ⅰ-Ⅱ	7×6×1.5cm	T3	N0	M0	3
65	2007.8	survive	60	female	76	no	Ⅰ-Ⅱ	7×6×4cm	T3	N0	M0	0
66	2007-10-18	survive	60	female	50	no	Ⅰ-Ⅱ	4×3×1cm	T3	N0	M0	3
67	2007.8	survive	60	female	72	no	Ⅰ-Ⅱ	4.5×4×1.5cm	T3	N0	M0	2
68	2007.6	death	40	female	69	no	Ⅰ-Ⅱ	7×5×1.5cm	T4a	N0	M0	2
69	2008.1	death	60	female	77	no	Ⅰ-Ⅱ	6×3×1cm	T3	N1a	M0	2
70	2007.9	death	17	female	67	no	Ⅰ-Ⅱ	2.5×2.3×0.6cm	T2	N0	M0	2
71	2008.4	death	36	female	75	no	Ⅰ-Ⅱ	5×4×1cm	T3	N0	M0	3
72	2007.6	survive	60	male	57	no	Ⅰ-Ⅱ	5×5×0.5cm	T3	N2a	M0	1
73	2007.6	survive	60	male	76	no	Ⅰ-Ⅱ	5×5×3cm	T3	N1b	M0	2
74	2007.9	survive	60	male	57	no	Ⅰ-Ⅱ	4.8×3.5×1.5cm	T2	N0	M0	3
75	2007-11-26	survive	60	male	90	no	Ⅰ-Ⅱ	6×5×2cm	T4b	N0	M0	3
76	2007-12-21	survive	60	male	67	no	Ⅰ-Ⅱ	5×4×1.5cm	T3	N0	M0	2
77	2008.1	survive	60	male	85	no	Ⅰ-Ⅱ	3×2.5×1cm	T3	N1a	M0	3
78	2008.2	survive	60	male	63	no	Ⅰ-Ⅱ	3×3×0.8cm	T3	N1b	M0	2
79	2008.3	survive	60	male	75	no	Ⅰ-Ⅱ	4.5×3×1cm	T3	N0	M0	3
80	2007-12-7	survive	60	male	73	no	Ⅰ-Ⅱ	6×5×1cm	T3	N0	M0	2
81	2007.6	death	47	male	91	no	Ⅰ-Ⅱ	4×4×1.3cm	T3	N0	M0	0
82	2007-12-4	death	31	male	72	no	Ⅰ-Ⅱ	9.5×6×2cm	T2	N0	M0	1

83	2008.3	survive	60	female	83	no	Ⅰ-Ⅱ	①7×5×2cm② 5×4×0.5cm		N1a	M0	2
84	2008.3	survive	60	female	80	no	Ⅰ-Ⅱ	4×6×5cm	T3	N0	M0	3
85	2007-11-1	survive	60	female	47	no	Ⅰ-Ⅱ	9×6×1cm	T3	N0	M0	1
86	2008.3	survive	60	female	86	no	Ⅰ-Ⅱ	9×6×1.5cm	T3	N0	M0	2
87	2008.4	survive	60	female	75	no	Ⅰ-Ⅱ	2.5×2×1cm	T4a	N2a	M0	3
88	2007-9-7	death	16	female	54	no	Ⅰ-Ⅱ	5×5×1cm，3×2.5×1cm	T4a	N1b	M0	3
89	2007-11-28	death	11	female	73	no	Ⅰ-Ⅱ	5×5×4cm	T3	N0	M0	3
90	2007.7	death	36	female	60	no	Ⅰ-Ⅱ	5×2×1.2cm	T3	N0	M0	0
91	2007.7	survive	60	male	61	no	Ⅰ-Ⅱ	6×4.5×1.5cm	T3	N1a	M0	3
92	2007.9	survive	60	male	65	no	Ⅰ-Ⅱ	7×4×1.5cm	T3	N0	M0	2
93	2008.4	survive	60	male	65	no	Ⅰ-Ⅱ	3.5×3×1cm	T3	N0	M0	2
94	2008.2	death	22	male	61	no	Ⅰ-Ⅱ	4×3×3cm	T3	N0	M0	1
95	2007.8	death	10	male	51	no	Ⅰ-Ⅱ	2.5×2×1.5cm	T4b	N1b	M0	1

Supplementary Table S2. Sequences of the Real-time PCR (all for human genes)

PT-qPCR Primers		
**Gene Name**	**Forward**	**Reverse**
**KDM6A**	GACATTGAGGGAAGCTCTCA	ACTTGCATCAGGTCCTCCAT
**KDM6B**	TGCTCCGTCAACATCAACAT	CTTCTGCTCCGTCAACATCA
**KDM5C**	GTGGTGGAGGAAGGTGGTTA	AGCGTAGCAAGGAGCCAATA
**CTNNB1**	ATGACTCGAGCTCAGAGGGT	ATTGCACGTGTGGCAAGTTC
**NOTCH1**	GCAGTTGTGCTCCTGAAGAA	CGGGCGGCCAGAAAC
**NOTCH2**	GGCATTAATCGCTACAGTTGTGTCT	GGAGGCACACTCATCAATGTCA
**HES7**	ACGGCCCCAAGATGCTCAA	TTTCTCCAGCTTCGGGTTCC
**JAG1**	GGAGGCGTGGGATTCCA	CCGAGTGAGAAGCCTTTCAATAAT
**CD133**	ACAATTCACCAGCAACGAGTCC	GACGCTTTGGTATAGAGTGCTCAG
**LGR5**	CTCCCAGGTCTGGTGTGTTG	GTGAAGACGCTGAGGTTGGA
**SOX2**	GTATCAGGAGTTGTCAAGGC	AGTCCTAGTCTTAAAGAGG
**NANOG**	TAGCAATGGTGTGACGCAGAAG	TCTGGTTGCTCCACATTGGAAGG
**CHIP- NOTCH2-P1**	AGATACAGGGACAGAAACA	GGAAATCCTACCCATAGA
**CHIP- NOTCH2-P2**	TCCGTCCTTGACTCCTCT	AACCTGGGTACAAGTGGG
**CHIP- NOTCH2-P3**	TCCATTCCTCTAAGGGTG	CCAAGACTTTGCCGCTGT
**CHIP- NOTCH2-P4**	CCACTCCGTGATGGCTCTA	CCGGGATCGTGAACTTGC
**CHIP- NOTCH2-P5**	GGGAGTCGAGGCATTTGCG	CCCTCAGCCCGATACTCACCAT
**CHIP- NOTCH2-P6**	GTTCCCAAGAGTTTGGACATCG	ATCTGGCATTCCTTCGCTCA
**CHIP- NOTCH2-P7**	GTCCAGACCCAGGAATGT	CTCCAAAGCCCAAGTTAT
**CHIP- NOTCH2-P8**	CCAGTGGGATGCTGTCTT	TCGGGTGATAAATAACCTTG

Supplementary Table S3. shRNA Sequences Targeting KDM6A/KDM6B

**shKDM6A**	Top strend	gatccGCTGTTCGCTGCTATGAATTTCAAGAGAATTCATAGCAGCGAACAGCTTTTTTACGCGTg
	Botton strend	aattcACGCGTAAAAAAGCTGTTCGCTGCTATGAATTCTCTTGAAATTCATAGCAGCGAACAGCg
**shKDM6B**	Top strend	gatccGATGATCTCTATGCATCCATTCAAGAGATGGATGCATAGAGATCATCTTTTTTg
	Botton strend	aattcAAAAAAGATGATCTCTATGCATCCATCTCTTGAATGGATGCATAGAGATCATCg

Annealed top and bottom strands were inserted into pLVX-shRNA1 plasmid. *Eco*RI and *Bam*HI were used as **restriction enzymes.**
